# Supplementary figures and images for: GAPDH mediates drug resistance and metabolism in Plasmodium falciparum malaria parasites
Source: PLoS Pathog. 2022 Sep 14;18(9):e1010803. doi: 10.1371/journal.ppat.1010803 (PMC9512246; doi:10.1371/journal.ppat.1010803)

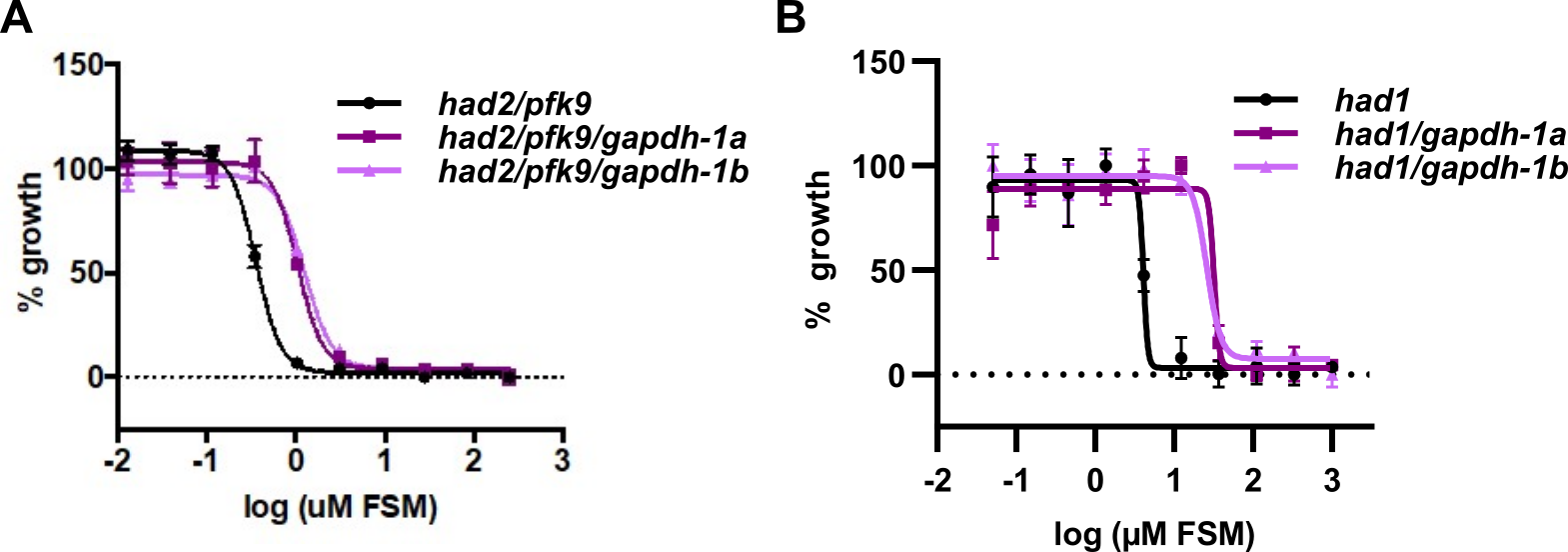

Supplement: S1 Fig — Representative dose-response curves (from n = 3 experiments), generated using nonlinear regression (GraphPad Prism). Half-maximal inhibitory concentrations (IC50s) for each strain are reported in S1 Table. (TIFF) [file ppat.1010803.s001.tiff]

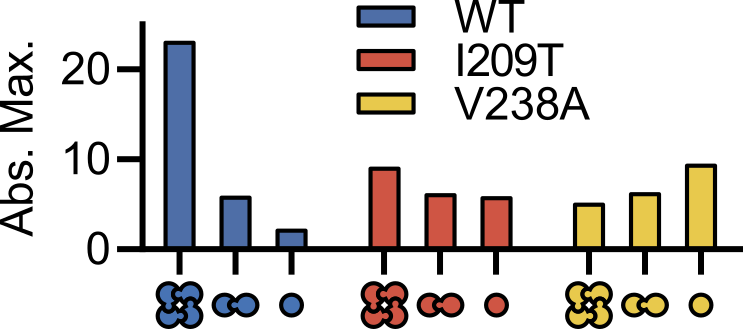

Supplement: S2 Fig — Absorbance maximums are reported for each of the peaks that correspond to the relative molecular weights for each oligomer. Tetramer, dimer, and monomer, illustrated by number of subunits as shown on the x-axis. (TIFF) [file ppat.1010803.s002.tiff]

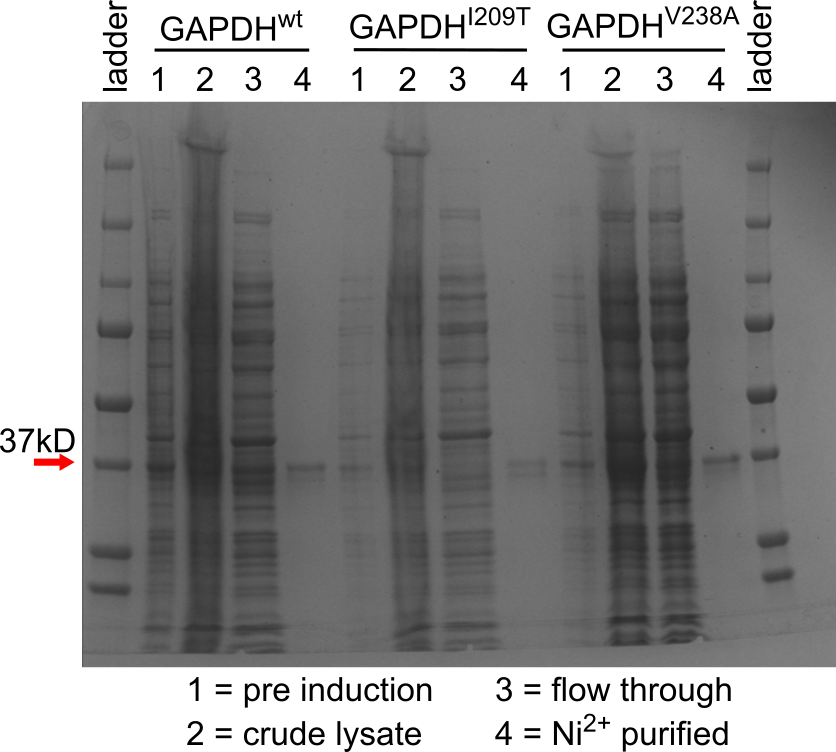

Supplement: S3 Fig — (TIFF) [file ppat.1010803.s003.tiff]

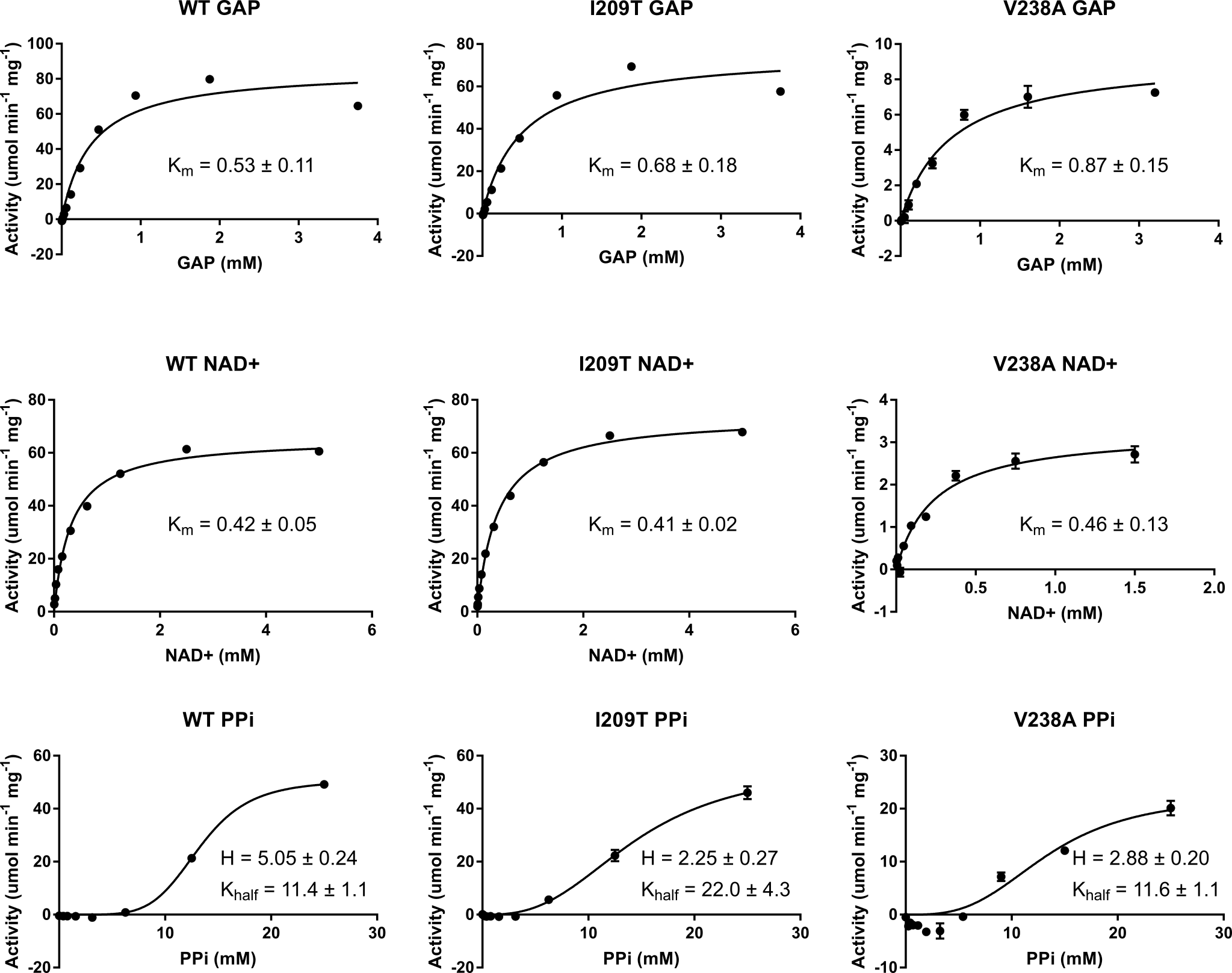

Supplement: S4 Fig — All curves are representative of experimental triplicates and calculations are derived from the non-linear regression function in GraphPad Prism. (TIFF) [file ppat.1010803.s004.tiff]

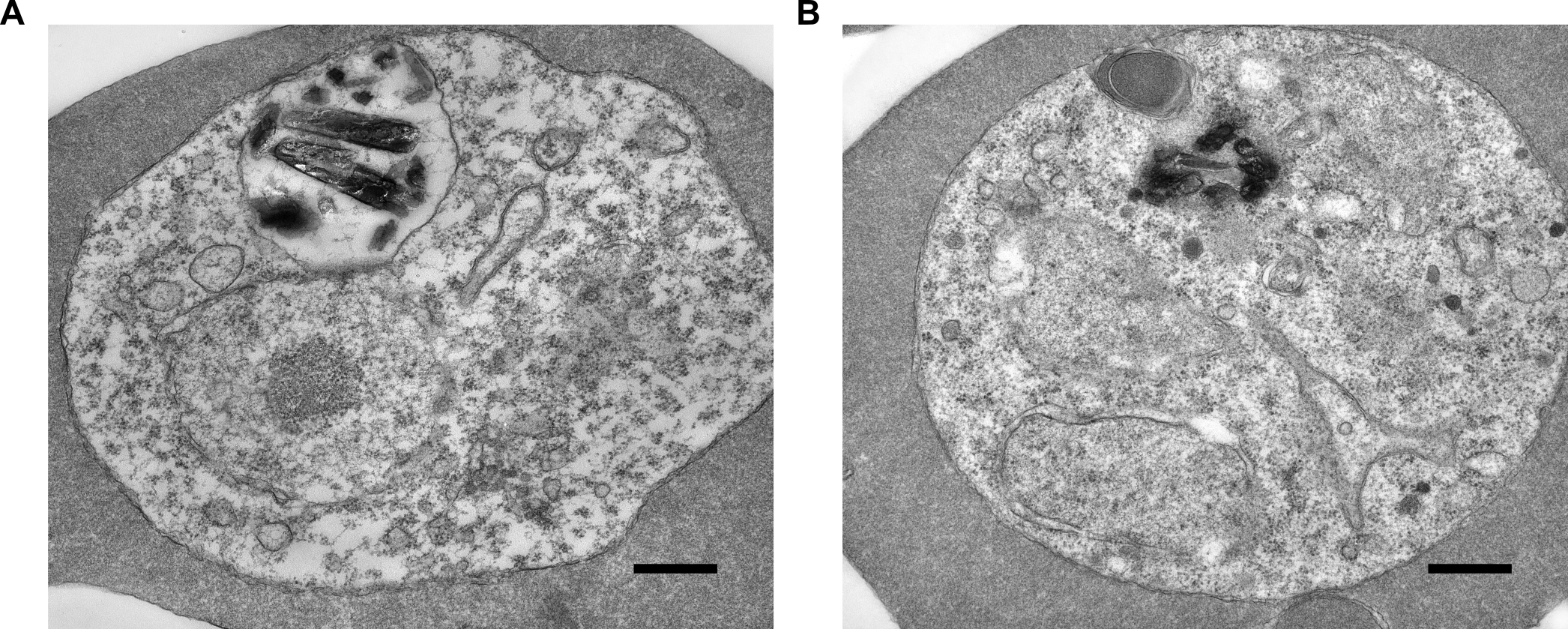

Supplement: S5 Fig — (A) Electron micrograph demonstrating the normal food vacuolar phenotype of P. falciparum trophozoites (DMSO treated), characterized by a low electron density vacuole containing dense hemozoin crystals. (B) Electron micrograph illustrating the food vacuole disruption following 12h of FSM treatment (5 μM). Hemozoin crystals are no longer contained by membrane and are now free within the cytosol. (TIFF) [file ppat.1010803.s005.tiff]

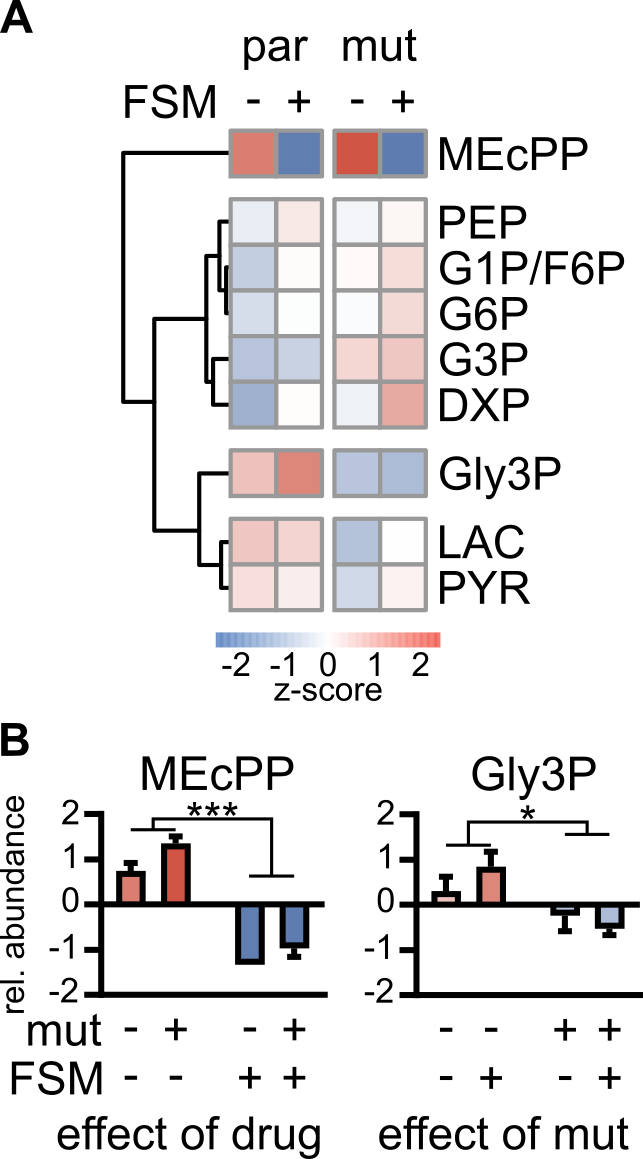

Supplement: S6 Fig — (A) Hierarchical clustering of relative abundances for targeted metabolites are displayed comparing parent (par) strains versus alternative gapdh alleles (mut) in the presence and absence of FSM treatment. Z-scores represent differences within metabolites across samples. (B) Type III two-way ANOVA for targeted metabolites only shows significant differences with respect to drug treatment for 2-C-methyl-D-erythritol-2,4,-cyclopyrophosphate (MEcPP) and with respect to genotype for glycerol 3-phosphate (Gly3P). (TIFF) [file ppat.1010803.s006.tiff]

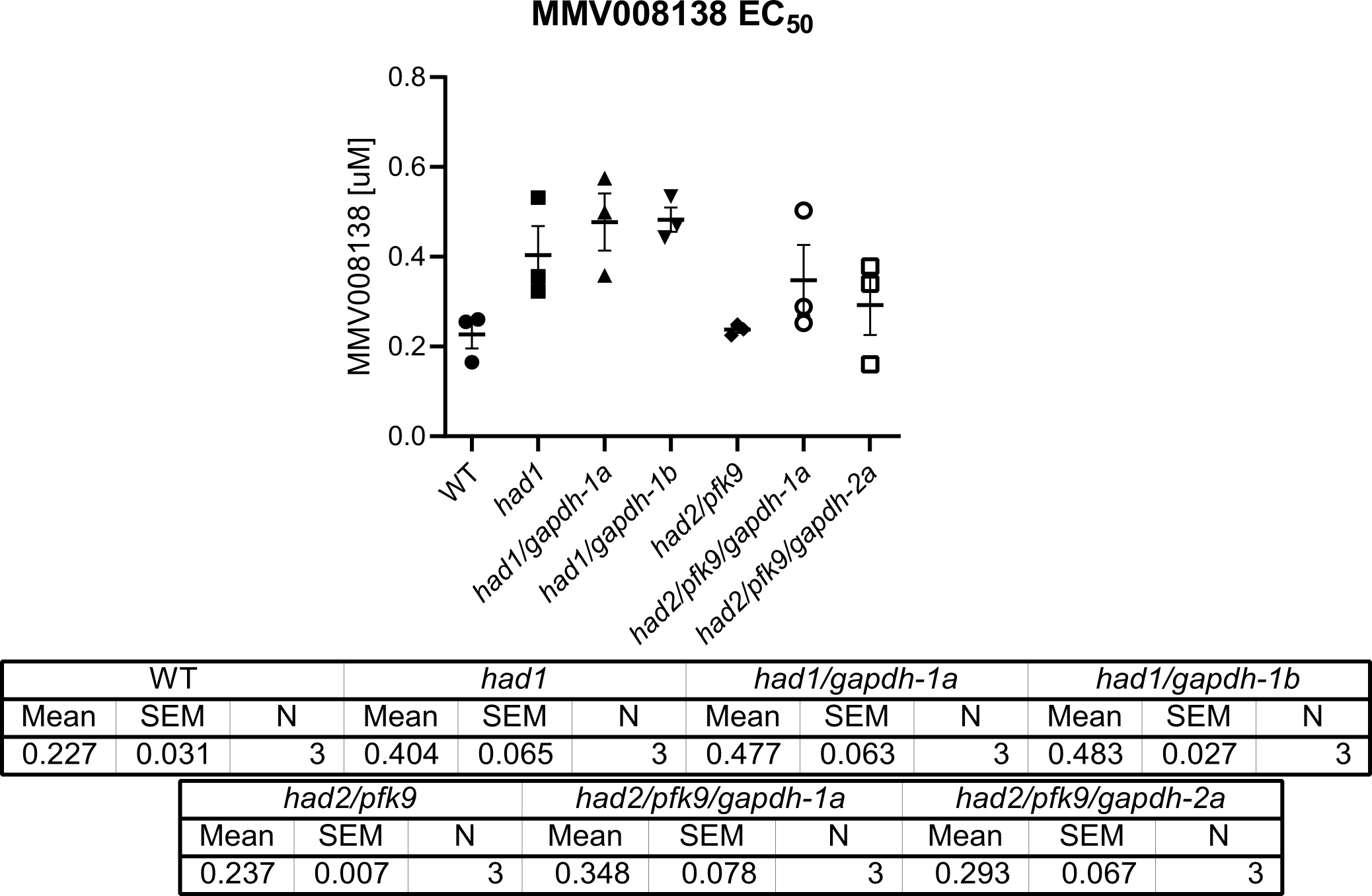

Supplement: S7 Fig — MMV008138 inhibits the MEP pathway enzyme IspD competitively with its CTP substrate. Summary EC50 data, determined using GraphPad Prism non-linear regression. (TIFF) [file ppat.1010803.s007.tiff]

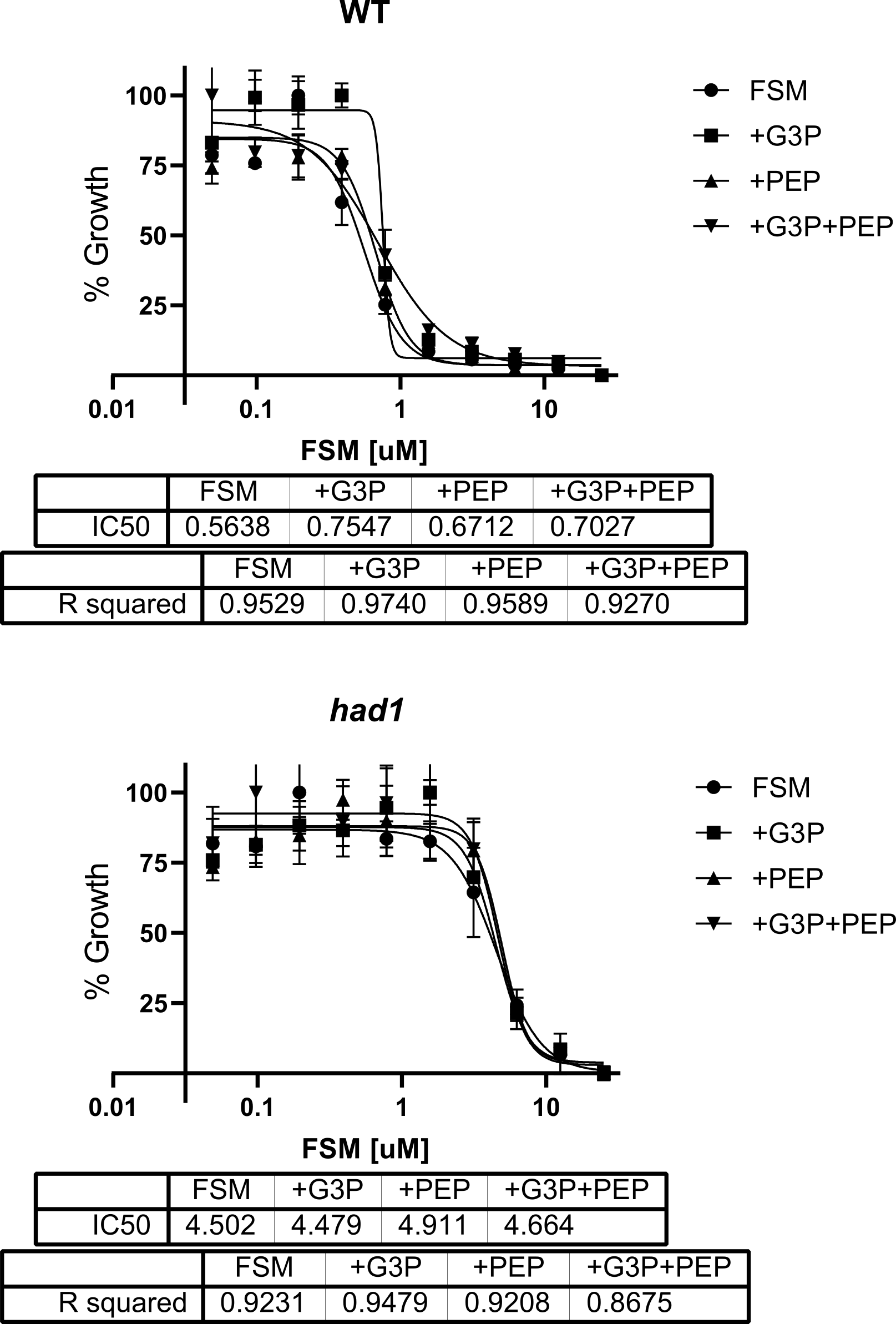

Supplement: S8 Fig — Exogenous glycolytic metabolites (G3P, glyceraldehyde 3 phosphate; PEP, phosphoenolpyruvate) were supplied at 100 μM. Top, 3D7 wild-type parasite strain. Bottom, had1 mutant parasite strain. EC50s were calculated using non-linear regression in GraphPad Prism. (TIFF) [file ppat.1010803.s008.tiff]

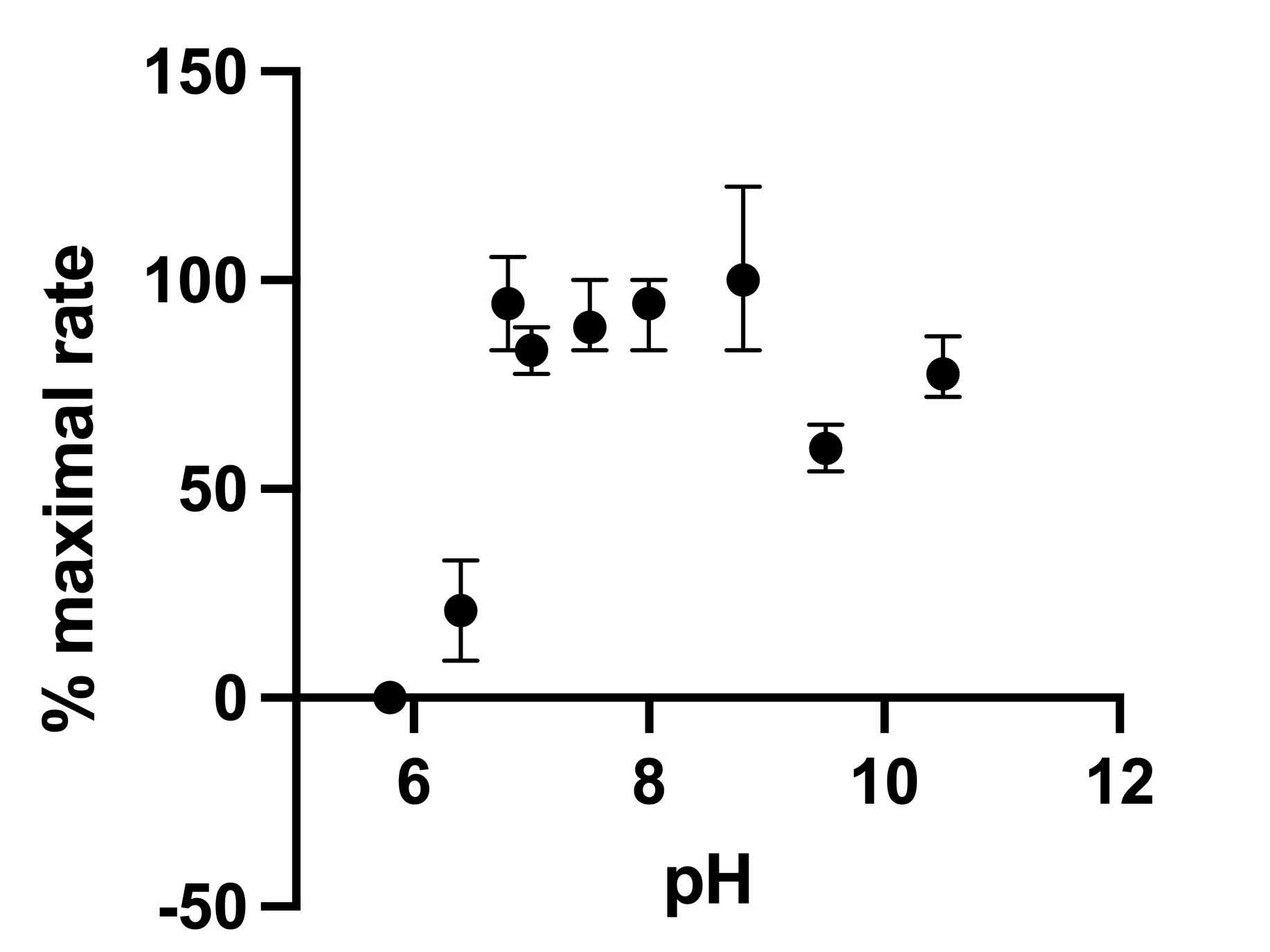

Supplement: S9 Fig — The enzymatic activity of purified P. falciparum GAPDH was quantified in buffers of increasing pH from 6 to 11, normalized to the maximal activity observed in all conditions. (TIFF) [file ppat.1010803.s009.tiff]
